# Supplementary figures and images for: Altered composition and functional profile of high-density lipoprotein in leprosy patients
Source: PLoS Negl Trop Dis. 2020 Mar 30;14(3):e0008138. doi: 10.1371/journal.pntd.0008138 (PMC7145193; doi:10.1371/journal.pntd.0008138)

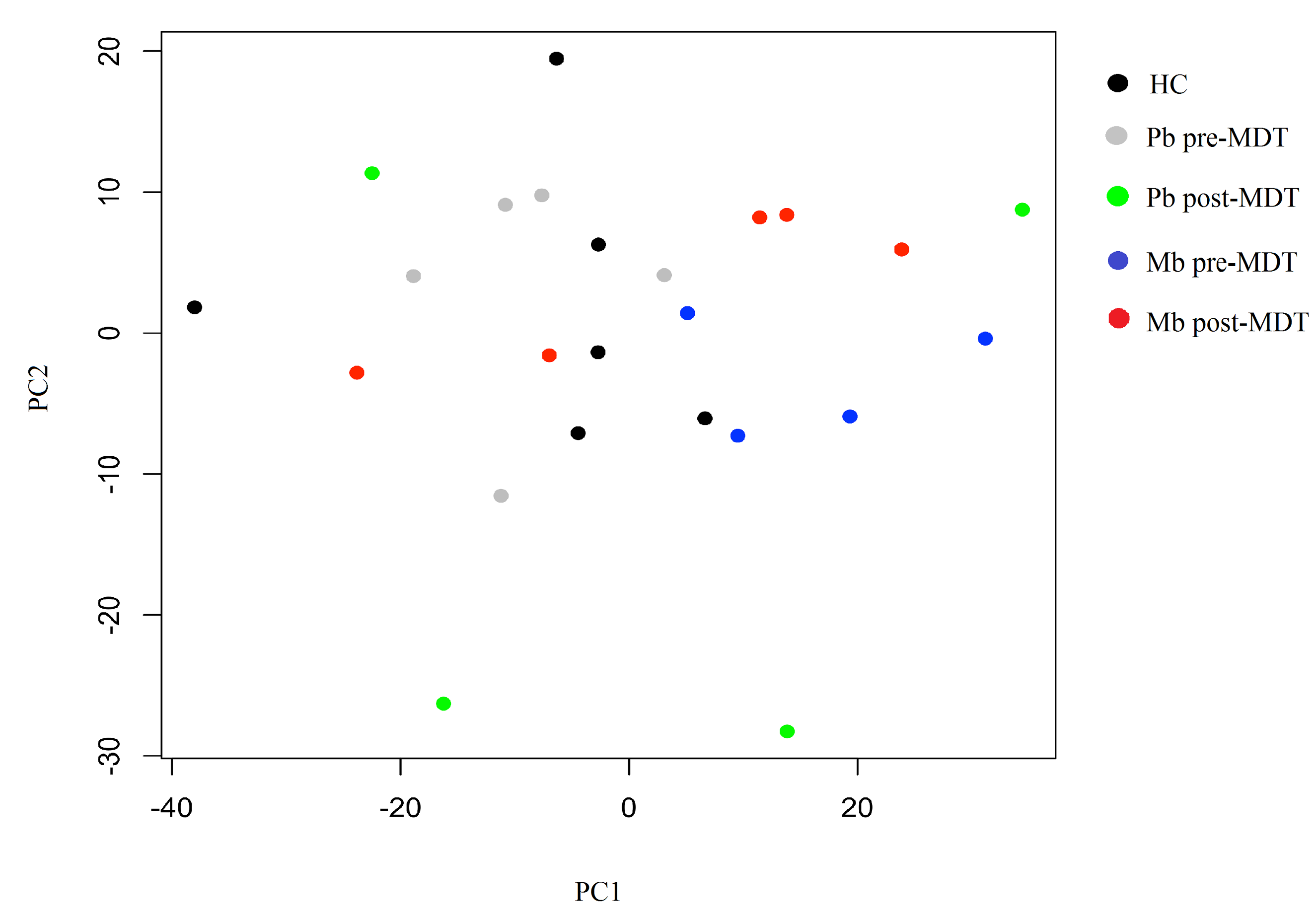

Supplement: S1 Fig — Raw UPLC-MS data collected in positive ionization mode were processed and analyzed by XCMS, followed by normalization through the quantile method. Subsequently, the molecular features (MFs) were grouped into spectra through RAMClustR. In this approach, each spectrum represents a “compound” with adducts, isotopes and monoisotopic mass. The intensities of 1260 “compounds” were used to perform a principal component analysis. HC (n = 6, black dot), Pb pre-MDT (n = 5, grey dot), Pb post-MDT (n = 4, green dot), Mb pre-MDT n = 4, blue dot), Mb post-MDT (n = 5, red dot). (TIF) [file pntd.0008138.s001.tif]

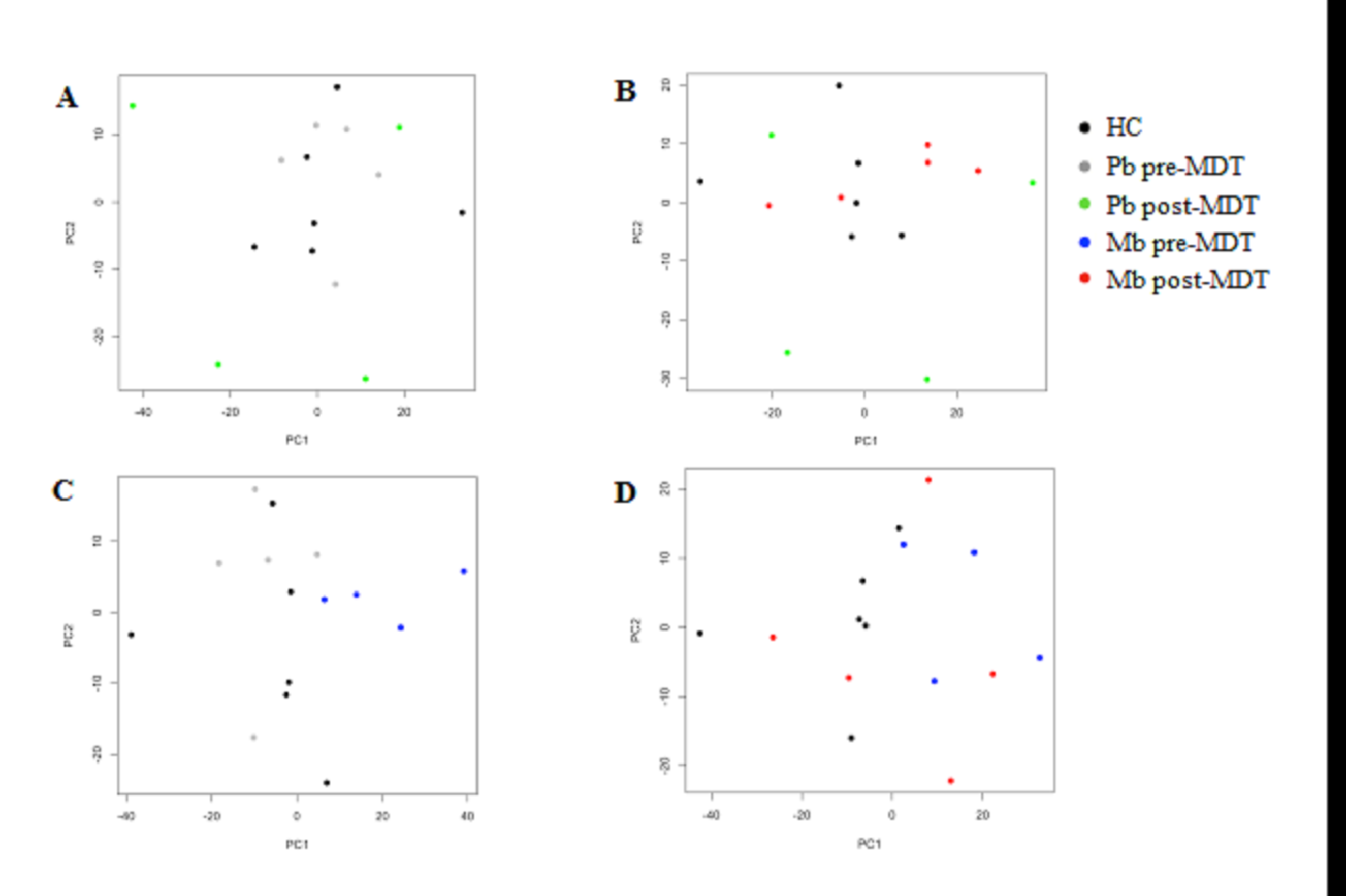

Supplement: S2 Fig — Raw UPLC-MS data collected in positive ionization mode were processed and analyzed by XCMS, followed by normalization through the quantile method. Subsequently, the molecular features (MFs) were grouped into spectra through RAMClustR. In this approach, each spectrum represents a compound group (named “compound”) with adducts, isotopes and monoisotopic mass. The intensities of 1260 “compounds” were used to perform a principal component analysis. (a) HC (n = 6, black dot), Pb pre-MDT (n = 5, grey dot) and Pb post-MDT (n = 4, green dot). (b) HC, Pb post-MDT and Mb post-MDT patients (n = 5 red dot). (c) HC individuals, Mb pre-MDT patients (n = 4, blue dot) and Pb patients. (d) HC, Mb patients and Mb post-MDT patients. (TIF) [file pntd.0008138.s002.tif]

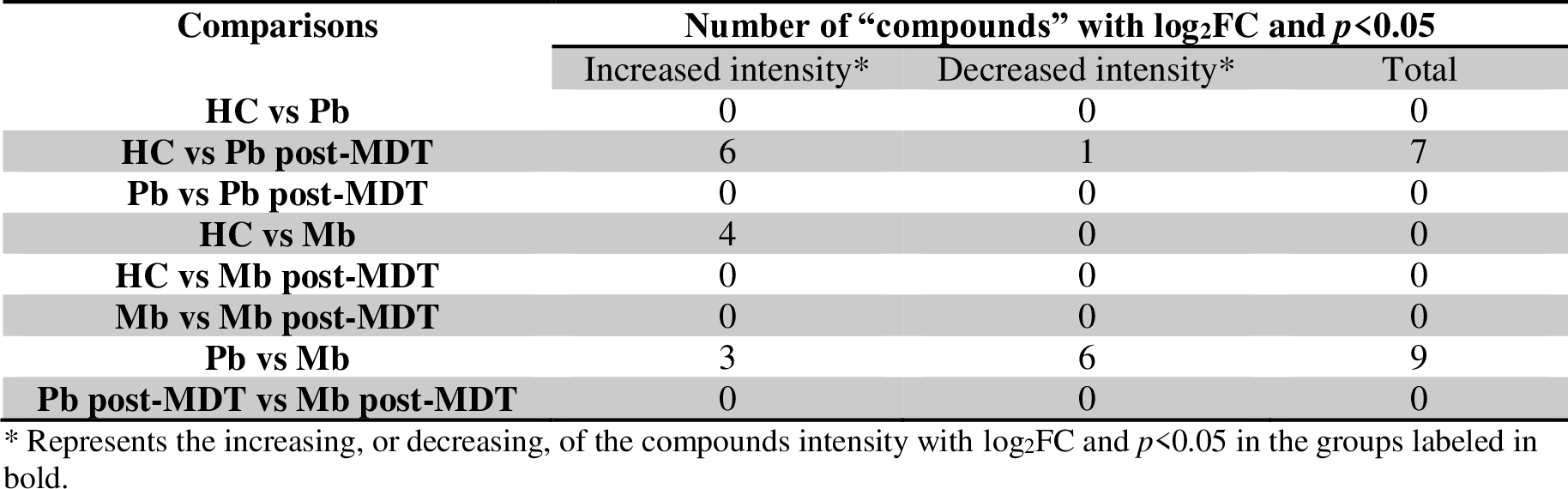

Supplement: S1 Table — (TIF) [file pntd.0008138.s004.tif]
