# Supplementary material for: Altered composition and functional profile of high-density lipoprotein in leprosy patients
Source: PLoS Negl Trop Dis. 2020 Mar 30;14(3):e0008138. doi: 10.1371/journal.pntd.0008138 (PMC7145193; doi:10.1371/journal.pntd.0008138)
Supplement: S2 Table — (PDF) [file pntd.0008138.s005.pdf]

S2 Table. Statistical comparison of the spectral entities ("compounds") with their respective log_2 fold change and *p* values.

	**HC vs Pb pre MDT***	
Compounds	Log_2FC	Adjusted *p* values
C83	+1.12	0.22
C501	-2.41	0.08
C543	+1.36	0.09
C698	-1.22	0.15
C916	-2.92	0.07
C945	-1.58	0.37
	**HC vs Pb post-MDT***	
Compounds	Log_2FC	Adjusted *p* values
C83	+1.73	$p<0.01$
C102	+1.32	$p<0.01$
C127	+1.26	0.01
C154	-2.28	0.05
C162	+1.29	$p<0.01$
C365	-2.35	0.05
C543	+1.88	$p<0.01$
C999	+1.35	$p<0.01$
C1020	-1.76	0.01
	**Pb pre-MDT vs Pb post-MDT***	
Compounds	Log_2FC	Adjusted *p* values
C127	+1.12	0.24
C154	-1.62	0.66
C211	-1.70	0.66
C296	3.08	0.66
C444	-1.46	0.66
C542	-1.34	0.66
C976	1.81	0.66
C1020	-1.50	0.32
	**HC vs Mb pre-MDT***	
Compounds	Log_2FC	Adjusted *p* values
C83	+1.74	0.01
C135	-1.12	0.23
C200	+1.10	0.04
C209	+1.25	0.01
C216	+2.60	0.23
C395	+1.96	0.23
C543	+1.74	0.01
	**HC vs Mb post-MDT***	
Compounds	Log_2FC	Adjusted *p* values
C131	+1.31	0.73
C164	+1.85	0.62
C363	-2.28	0.62
C501	-2.21	0.15
C916	-2.59	0.13
	**Mb pre-MDT vs Mb post-MDT***	
Compounds	Log_2FC	Adjusted *p* values
C83	-1.21	0.38
C164	+2.87	0.20
C209	-1.02	0.20
C216	-2.59	0.38
C493	-2.72	0.38
C501	-2.02	0.38
C526	-2.15	0.38
C916	-2.36	0.38
C975	+2.74	0.38
	**Pb pre-MDT vs Mb pre-MDT ***	
Compounds	Log_2FC	Adjusted *p* values
C200	+1.15	0.03
C209	+1.42	$p<0.01$
C273	-2.36	0.04
C563	+1.15	0.03

C608	-1.54	0.04
C774	-1.57	0.03
C835	-1.55	0.03
C1062	-1.45	0.04
C1065	-2.42	0.03
**Pb post-MDT vs Mb post-MDT***		
Compounds	Log_2FC	Adjusted *p* values
C44	+1.42	0.20
C151	+2.84	0.15
C154	+2.61	0.08
C164	+2.93	0.08
C267	+1.37	0.14
C365	+2.21	0.20
C371	+1.90	0.15
C491	+1.66	0.20
C936	+1.41	0.20
C1020	+1.39	0.20

In this table we only show the "compounds" that exhibit a $log_2FC \geq 1.0$. The spectral intensity for each "compound" (or only intensity) was considered statistically significant when log_2FC value was ≥1.0 and the adjusted *p* value was <0.05. The negative (-) signal represents that the intensity of the "compound" was decreased in the group with asterisk (*), while the positive signal (+) means that the intensity of the "compound" was increased. HC (n=6); Pb pre-MDT (n=5); Pb post-MDT (n=4); Mb pre-MDT (n=4); Mb post-MDT(n=5). HC: healthy controls; Pb: paucibacillary patients; Mb: multibacillary patients; Pre-MDT: before treatment with multidrug therapy; Post-MDT: after treatment with multidrug therapy.
