# Supplementary material for: Altered composition and functional profile of high-density lipoprotein in leprosy patients
Source: PLoS Negl Trop Dis. 2020 Mar 30;14(3):e0008138. doi: 10.1371/journal.pntd.0008138 (PMC7145193; doi:10.1371/journal.pntd.0008138)
Supplement: S3 Table — (PDF) [file pntd.0008138.s006.pdf]

S3 Table. Putative identification of the "compounds" with $log_2FC \geq 1.0$ and $p<0.05$

"Compound"	Query Mass	Formula	Compound Mass	Adduct	Adduct Mass	Delta	Lipid ID
C83	119.09	$C_{20}H_{34}O_5$	354.24	M+3H	119.09	0.00168	13,14-Dihydro-lipoxin A4†
							Prostaglandin D1†
							11b-PGF2a†
							11,12,15-TriHETRE†
							11-Epi-PGF2a†
							8-Isoprostaglandin F2a†
							11,14,15-THETA†
							Prostaglandin E1†
							Prostaglandin F2a†
							Prostaglandin F2b†
							Trioxilin B3†
							Trioxilin A3†
							8-isoprostaglandin PGF2b†
							11,12,15-THETA†
							13,14-Dihydro-15-keto PGF2a†
							8-Isoprostaglandin E1†
							8-iso-13,14-dihydro-15-keto-PGF2a†
							Prostaglandin H1†
							15-keto-PGF1alpha†
							(5Z,9E,12S,14Z)-8,11,12-Trihydroxyicosa-5,9,14-trienoate†
	415.21	$C_{20}H_{34}O_6$	370.23	M+2Na-H	415.21	0.0055	10,11-dihydro-20-dihydroxy-LTB4*
							Prostaglandin G1*
							Thromboxane B2*
							6-Keto-prostaglandin F1a*
							20-Hydroxy-PGF2a*
C102	119.09	$C_{20}H_{34}O_5$	354.24	M+3H	119.09	0.00128	Prostaglandin E1†
							11b-PGF2a†
							11,12,15-TriHETRE†
							11-Epi-PGF2a†
							8-iso-13,14-dihydro-15-keto-PGF2a†
							Prostaglandin D1†
							8-Isoprostaglandin F2a†
							Prostaglandin F2a†
							Prostaglandin F2b†
							Trioxilin B3†
							Trioxilin A3†
							8-isoprostaglandin PGF2b†
							11,12,15-THETA†
							13,14-Dihydro-15-keto PGF2a†
							8-Isoprostaglandin E1†
							11,14,15-THETA†
							Prostaglandin H1†
							15-keto-PGF1alpha†
							(5Z,9E,12S,14Z)-8,11,12-Trihydroxyicosa-5,9,14-trienoate†
							13,14-Dihydro-lipoxin A4†
C127	331.13	$C_{18}H_{22}O_3$	286.15	M+2Na-H	331.13	0.00335	17beta-Estradiol-2,3-quinone
							17beta-Estradiol-3,4-quinone

							16a-Hydroxyestrone
							2-Hydroxyestrone
							16-Ketoestradiol
							16b-Hydroxyestrone
							4-Hydroxyestrone
	387.19	$C_{21}H_{32}O_4$	348.23	M+K	387.19	0.00028	17a,21-Dihydroxypreg-nenolone†
							11b,21-Dihydroxy-5b-pregnane-3,20-dione†
							3a,21-Dihydroxy-5b-pregnane-11,20-dione†
							3b,15b,17a-Trihydroxy-pregnenone†
C162	147.11	$C_{26}H_{46}O_5$	438.33	M+3H	147.11	0.000884	27-Nor-5b-cholestane-3a,7a,12a,24,25-pentol†
	282.20	$C_{33}H_{54}O_7$	562.38	M+2H	282.20	0.003747	Cholesterolglucuronide
	283.20	$C_{28}H_{54}NO_7P$	547.36	M+H+NH4	283.20	0.005355	LysoPC(20:2(11Z,14Z))*
C200	102.09	$C_{18}H_{35}NO$	281.27	M+2H+Na	102.09	0.000178	Oleamide†‖
	284.29	$C_{18}H_{37}NO$	283.28	M+H	284.29	0.000609	Octadecanamide‡
	306.27	$C_{18}H_{37}NO$	283.28	M+Na	306.27	0.000467	Octadecanamide‡
C209	256.26	$C_{16}H_{33}NO$	255.25	M+H	256.26	0.000509	Palmiticamide‡
	278.24	$C_{16}H_{33}NO$	255.25	M+Na	278.24	0.000267	Palmiticamide†‡
	102.09	$C_{18}H_{35}NO$	281.27	M+2H+Na	102.09	0.000378	Oleamide‖
C273	594.38	$C_{18}H_{24}O_3$	288.17	2M+NH4	594.38	0.00161	Estriol†
							4-hydroxystradiol†
							2-Hydroxyestradiol†
							16b-Hydroxyestradiol†
							17-Epiestriol†
							16,17-Epiestriol†
							4-Hydroxyestradiol†
		$C_{60}H_{112}N_2O_{18}$	114.87	M+H+K	594.38	0.003424	Ganglioside GA2 (d18:1/22:0)
		$C_{29}H_{57}NO_9P$	593.36	M+H	594.37	0.0008	PC(16:0/5:0(CHO)), POVPC‡a
	595.38	$C_{19}H_{26}O_2$	286.19	2M+Na	595.37	0.005422	Androstenedione*
	616.35	$C_{29}H_{57}NO_9P$	593.36	M+Na	616.35	0.0005	PC(16:0/5:0(CHO)), POVPC†‡a
	668.41	$C_{67}H_{122}N_2O_{21}$	129.08	M+2Na	668.41	0.003422	Ganglioside GM3 (d18:1/26:1(17Z))
		$C_{62}H_{112}N_2O_{23}$	125.27	M+2ACN+2H	668.42	0.00382	Tetrahexosylceramide (d18:1/9Z-18:1)
							Ganglioside GA1 (d18:1/9Z-18:1)
C543	-	-	-	-	-	-	Unidentified
C563	250.21	$C_{42}H_{86}NO_7P$	747.61	M+3H	250.21	0.002794	PC(o-16:0/18:0)□
C608	701.55	$C_{42}H_{78}O_5$	662.58	M+K	701.54	0.001684	DG(20:0/0:0/18:2n6)
		$C_{20}H_{37}NO_3$	339.27	2M+Na	701.54	0.002494	Oleoylglycine
	743.56	$C_{47}H_{76}O_5$	720.56	M+Na	743.55	0.001106	DG(22:6(4Z,7Z,10Z,13Z,16Z,19Z)/22:2(13Z,16Z)/0:0) †
		$C_{45}H_{78}O_5$	698.58	M+2Na-H	743.55	0.003514	DG(18:4(6Z,9Z,12Z,15Z)/24:1(15Z)/0:0)
		$C_{46}H_{82}O_2$	666.63	M+2K+H	743.55	0.009078*	Campesterylstearate
							CE(19:0)
C774	159.12	$C_{27}H_{48}O_5$	452.35	M+2H+Na	159.12	0.00058	5a-Cholestane-3a,7a,12a,23,25-pentol†
							5b-Cholestane-3a,7a,12a,24,25-pentol†
							5b-Cholestane-3a,7a,12a,23R,25-pentol†
							5b-Cholestane-3a,7a,12a,25,26-pentol†

							Cholestane-3,7,12,24,25-pentol†
							5b-Cholestane-3a,7a,12a,23S,25-pentol†
C835	351.20	$C_{18}H_{32}O_5$	328.22	M+Na	351.21	0.005192	2,3-Dinor-8-iso prostaglandin F1alpha*
	735.55	$C_{49}H_{76}O_2$	696.58	M+K	735.54	0.00351	CE(22:6(4Z,7Z,10Z,13Z,16Z,19Z) †‡
	741.54	$C_{49}H_{76}O_2$		M+2Na-H	741.55	0.012492	CE(22:6(4Z,7Z,10Z,13Z,16Z,19Z)) ‡*
C999	-	-	-	-	-	-	Unidentified
C1020	158.15	$C_{18}H_{35}NO_2$	297.26	M+H+NH4	15.81	0.00026	Palmitoleoyl ethanolamide
C1062	145.10	$C_{23}H_{44}NO_2$	366.33	M+3Na	14.51	0.00032	Alpha-Linoleoylcholine
C1065	383.33	$C_{27}H_{42}O$	382.32	M+H	383.33	0.00076	7-Dehydrodesmosterol†
							Cholesta-4,6-dien-3-one†
							5a-Cholesta-8,24-dien-3-one†
		$C_{42}H_{86}NO_7P$	747.61	M+H+N H_4	383.32	0.003955	PC(o-16:0/18:0)□
		$C_{20}H_{39}NO_3$	341.29	M+ACN +H	383.32	0.004783	Stearoylglycine‡
	705.57			2M+Na	705.57	0.002494	Stearoylglycine‡

† Compounds with the smallest delta in the group. ‡Compounds with two different adducts in the same group. ǀCompounds present in more than one group (same adduct). □ Compounds present in more than one group (different adducts). *Delta > 0.005. **a** identified in the LIPIDS MAPS database, the others were identified by the HMDB.
