# Supplementary material for: Altered composition and functional profile of high-density lipoprotein in leprosy patients
Source: PLoS Negl Trop Dis. 2020 Mar 30;14(3):e0008138. doi: 10.1371/journal.pntd.0008138 (PMC7145193; doi:10.1371/journal.pntd.0008138)
Supplement: Attachment — Submitted filename: Response to reviewers.docx [file pntd.0008138.s007.docx]

Response to reviewers

We listed below ‘Editorial PLOS NTD’ questions, reviewer considerations that needed to be addressed (bold) and the authors’ replies (italics).

Response to reviewer #1:

1) Are the objectives of the study clearly articulated with a clear testable hypothesis stated? Reviewer # 1 consideration: “**hypothesis was not clearly articulated with a testable”.**

*The hypothesis was revised according to tests performed, as highlighted on the manuscript.*

2) Is the sample size sufficient to ensure adequate power to address the hypothesis being tested? Reviewer # 1 consideration: **“low number of patients but proof of principle study”.**

*Our data were based in a low number of cases, mainly due to the complex process of HDL purification. Despite the low number, data analysis showed a significant difference between Mb patients and healthy controls, which may be due to data homogeneity.*

3) Were correct statistical analysis used to support conclusions? Reviewer # 1 consideration: **“no statistical analysis”**

*Statistical analysis was revised. More suitable tests to the available sample number were applied, as highlighted on the manuscript.*

4) Are the conclusions supported by the data presented? Reviewer # 1 consideration: “**conclusions are supported by the data except the low number of cases as written above. but this can be considered as a fundamental research study showing what to search for in the future.”**

*We thank for the positive comment and improved the conclusion, adding perspectives for future research, as highlighted on the manuscript.*

5) Are the limitations of analysis clearly described? Reviewer # 1 consideration: **“Limitations could be more described”.**

*Main limitations were because of technical difficulties to obtain purified HDL, which were highlighted on the manuscript.*

6) Is public health relevance addressed? Reviewer # 1 consideration **“public relevance could be addressed.”**

*Public relevance was addressed on the conclusion.*

7) Editorial and Data Presentation Modifications? Reviewer # 1 consideration: **Shortening the material and methods since it is more than 8 pages.**

*We shortened the “Material and methods” session from eight to six pages.*

8) Minor comments related to sentences: **Lines 519-521: revise the sentence and add the number of patients involved in this Brasilian cohort.**

*The sentence was revised and highlighted on the manuscript.*

9) Major comments 1. Reviewer # 1 consideration: **Due to low numbers, it could have been more relevant to distinguish three studies, one with MB compared to HC, one comparing MB to PB and one comparing before and after MDT.**

*Graphics of Fig 1, 4 and 5 were separated for better comparisons between groups HC, Mb and Pb. A supplementary figure (S3 Fig) was created to add the comparisons between patients before and after MDT.*

10) Major comments 2. Reviewer # 1 consideration: **“How did the authors calculate the statistical differences?”.**

*Considering that all groups have a low number of cases and presented non-normal distributions, statistical differences observed on assays were calculated applying Mann-Whitney or Kruskall-Wallis tests.*

11) Major comments 3: Reviewer # 1 consideration: **Moreover, the patients after MDT are not the same as those before MDT. So interpatient comparison is difficult to assess.**

*It was not possible to make interpatient comparisons because it is difficult to obtain paired patient samples. Therefore, only samples from different patients (Mb or Pb before or after MDT) were available to perform the study. Mann-Whitney test was applied to analyze pre-MDT and post-MDT groups, as highlighted on the manuscript.*

*The authors thank reviewer#1 for the positive comments about the manuscript, and for the thorough evaluation of the work.*

___________________________________________________________________________

Response to reviewer #2:

1) Is the population clearly described and appropriate for the hypothesis being tested? Reviewer #2 consideration: **The definitions of healthy controls and post-MDT participants were not clearly described.**

*We replied reviewer #2 with the follow sentence added on the manuscript: “Pb post-MDT patients correspond to those who were treated with dapsone and rifampicin for six months, and Mb post-MDT patients were treated with dapsone, rifampicin and clofazimine for twelve months, according to the criteria established by WHO [26, 27]. Both Mb and Pb patients included in pre-MDT or post-MDT groups are different individuals. All participants were non-smokers and leprosy patients had no diagnosed comorbidities. Leprosy patients were recruited from “Souza Araújo” Leprosy Outpatient Unit (Oswaldo Cruz Foundation, Rio de Janeiro, RJ, Brazil). Healthy controls, all residents in the city of Rio de Janeiro (State of Rio de Janeiro, Brazil), were selected according to the similarity of age and gender patient’s cohort, and weren’t contacts of leprosy patients.”*

2) Is the sample size sufficient to ensure adequate power to address the hypothesis being tested? Reviewer #2 consideration: **It was also unclear how the sample size was determined.**

*Our data were based in a low number of cases, mainly due to the complex process of HDL purification. Despite the low number, data analysis showed a significant difference between Mb patients and healthy controls, which may be due to data homogeneity.*

3) Minor comments related to sentences: Reviewer #2 consideration: **Sentence modification on lines: 105-109, 107-109, 122-124, 131 213, 263, 285 and 536.**

*The requested modifications were performed and highlighted on the manuscript.*

4) Major comments: Reviewer #2 consideration: **It would also be better if the authors can provide more information on how the total sample size of 39 was determined.**

*The number of samples was limited because patient attendance to the outpatient clinic is scarce. It was necessary to use freshly collected plasma, in specific dates between diagnosis or end of MDT, and HDL separation needed to be performed in a careful and longstanding manner, to prevent endotoxin contamination or protein oxidation. Despite the low number of samples, data analysis showed a significant difference between Mb patients and healthy controls, probably due to data homogeneity.*

*The authors thank reviewer#2 for the positive comments about the manuscript, and for the thorough evaluation of the work.*
